# Many ways to break a heart

**DOI:** 10.7554/eLife.10040

**Published:** 2015-08-25

**Authors:** Megan Rowton, Ivan P Moskowitz

**Affiliations:** Departments of Pediatrics, Pathology and Human Genetics, University of Chicago, Chicago, United States; Departments of Pediatrics, Pathology and Human Genetics, University of Chicago, Chicago, United Statesimoskowitz@uchicago.edu

**Keywords:** cardiogenesis, transcription factors, genetic diseases, mouse

## Abstract

A mutant transcription factor that has been linked to congenital heart disease has wider effects than previously thought.

**Related research article** Bouveret R, Waardenberg AJ, Schonrock N, Ramialison M, Doan T, de Jong D, Bondue A, Kaur G, Mohamed S, Fonoudi H, Chen CM, Wouters M, Bhattacharya S, Plachta N, Dunwoodie SL, Chapman G, Blanpain C, Harvey RP. 2015. NKX2-5 mutations causative for congenital heart disease retain functionality and are directed to hundreds of targets. *eLife* **4**:e06942. doi: 10.7554/eLife.06942

**Image** Mutant forms of the NKX2-5 transcription factor are still able to influence how many genes are expressed

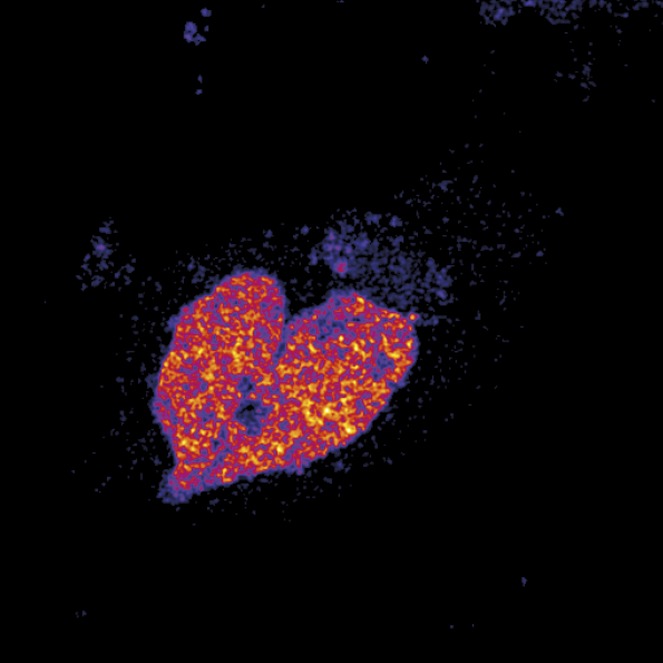

Congenital heart disease affects at least 1 in every 100 people worldwide, making these developmental defects and their consequences a significant clinical burden (Gelb and Chung, 2014). While the gene regulatory networks that control cardiac development and disease have been studied for many years (Vincent and Buckingham, 2010; Waardenberg et al., 2014; Paige et al., 2015), the field has yet to mechanistically link disruptions of cardiac gene networks to the pathology of congenital heart disease.

Proteins called transcription factors bind to specific DNA sequences, such as promoters and enhancers, to control gene expression. Cardiogenic transcription factors, such as NKX2-5, TBX5 and GATA4, play critical roles in ensuring that the embryonic heart forms properly, and dominant mutations in the genes that encode these transcription factors have been implicated in the development of congenital heart defects. A commonly-held assumption is that disease-associated mutations in the DNA binding region of transcription factors act primarily to decrease the functionality of the protein, thus altering the expression levels of their target genes. Now, in eLife, researchers at the Victor Chang Cardiac Research Institute, the University of New South Wales, the Université Libre de Bruxelles and other institutions in Australia, Belgium, Iran and the UK – including Romaric Bouveret as corresponding author and Richard Harvey as last author – challenge this assumption for the cardiogenic transcription factor NKX2-5 (Bouveret et al., 2015).

NKX2-5 was originally described in the fruit fly *Drosophila* where it was named *tinman,* as without it, the heart fails to form (Bodmer, 1993). Two decades ago Harvey and co-workers first described the heart defects caused by the inactivation of the mouse *Nkx2-5* gene (Lyons et al., 1995), and have been leaders in investigating the role of NKX2-5 and how it functions in cardiac biology since (Stanley et al., 2002; Elliott et al., 2006; Prall et al., 2007; Watanabe et al., 2012; Behrens et al., 2013). Now Bouveret et al. have analyzed how the mutations in NKX2-5 that are associated with congenital heart disease affect cardiac and non-cardiac gene regulatory networks.

Bouveret et al. used a technique called DamID to detect sites where NKX2-5 binds to DNA in cardiac muscle cells. Two mutant versions of NKX2-5 were also investigated: one, called NKX2-5Y191C, harbors a mutation located within the homeodomain – the region of NKX2-5 that binds to DNA – that has been linked to congenital heart disease; and another that completely lacks the homeodomain.

Neither mutation prevented NKX2-5 from binding to all of its usual targets. In fact, both NKX2-5 mutants were able to bind to and repress transcription from a known NKX2-5 regulatory element that controls the expression of *Id3,* a gene that inhibits cardiomyocyte differentiation. Overexpressing NKX2-5Y191C in cardiac progenitor cells also led to normal levels of activation and repression of some NKX2-5 target genes.

Transcription factors often bind to other proteins and molecules – known as cofactors – that regulate their activity. Bouveret et al. showed that the mutant forms of NKX2-5 maintained contact with non-mutant copies of NKX2-5 and other co-factors. This finding suggests that the effect of transcription factor mutations may be ameliorated because the proteins retain the ability to form dimers, and provides a model that may partially explain why some human patients with NKX2-5 homeodomain mutations exhibit only mild symptoms.

Most strikingly, Bouveret et al. have identified hundreds of DNA sites that the mutant NKX2-5 proteins bind to, but which are not targeted by wild-type NKX2-5. A technique called motif analysis implicated members of a broadly expressed protein family, known as the ETS transcription factors, as potential binding partners for the NKX2-5 mutants. Bouveret et al. further demonstrated that ETS family members, such as ELK1 and ELK4, act as co-factors for both wild-type and mutant NKX2-5 proteins within the cardiac gene regulatory network, but that this interaction is enriched in the homeodomain mutants.

Bouveret et al. conclude that interactions with ETS co-factors guide NKX2-5 mutants to a large set of “off-target” genes – perhaps the homeodomain mutants lose some of their DNA binding specificity, allowing co-factors such as ETS to play a stronger role in determining a new pattern of binding. Importantly, NKX2-5 mutants were shown to regulate the expression of these off-target genes, which together could be viewed as a “mutation-acquired gene regulatory network.” Whereas wild-type NKX2-5 tended to target genes important for heart development and physiology, the mutant forms of NKX2-5 targeted genes involved in the regulation of more general cellular processes, such as chromatin organization and cell cycle control (Figure 1). These mutant-specific targets may include genes that contribute to the developmental defects and heart pathologies observed in patients with NKX2-5 homeodomain mutations.

**Figure 1.**
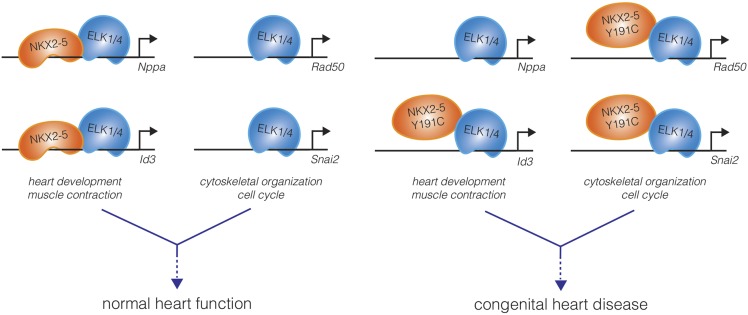
A mutation in the DNA-binding domain of NKX2-5, associated with congenital heart disease, leads to off-target effects in cardiomyocytes. In the wild-type state (left), the NKX2-5 DNA-binding domain directs the specificity of DNA binding of the NKX2-5:ELK1/4 complex to cardiac lineage-specific genes, such as *Nppa* and *Id3*. ELK1/4 also binds to genes involved in generic cellular processes, such as cytoskeletal organization and cell cycle control, but this regulation does not involve NKX2-5. The total of these interactions, and others, direct normal heart development and physiology. Bouveret et al. found that a mutation in the NKX2-5 DNA-binding domain (NKX2-5 Y191C) inhibits the direct binding of NKX2-5 to cardiac genes (right). This results in a loss of NKX2-5 localization at some cardiac genes (*Nppa*), while occupancy at other genes is maintained indirectly (*Id3*). Additionally, NKX2-5 binding to ELK1/4 is preserved, and the NKX2-5 Y191C:ELK1/4 complex is now directed to generic ELK1/4 targets, perhaps leading to the misregulation of these genes in cardiomyocytes. The disordered gene expression caused by the combination of loss-of-function and gain-of-function effects may underlie the origins of pathologies such as congenital heart disease.

The results presented by Bouveret et al. raise important questions about the mutations associated with congenital heart disease and provide fertile ground for future studies. It will be important to understand the degree to which the mutation-acquired gene regulatory network causes mutation-associated heart problems. This work potentially provides a novel paradigm for how congenital heart disease results from transcription factor mutations, and should encourage the field to examine the combined effects of both misregulated off-targets and misregulated wild-type targets when considering the effects of these mutations.

Of broad importance is whether the off-target binding of NKX2-5 mutants represents a generalizable phenomenon – do mutations in the DNA-binding domains of other highly specific transcription factors lead to the regulation of new targets via interactions with cofactors? This work highlights the importance of interactions between lineage-specific cardiac transcription factors (e.g., NKX2-5) and more broadly expressed co-factors (e.g., ETS) in the regulation of gene expression. Furthermore, it suggests that the specific control of cardiac regulatory genes is embedded within a broader and more ancient network that regulates ubiquitous cellular processes.
